# Store-Operated Ca^2^^+^ Entry (SOCE) Regulates Melanoma Proliferation and Cell Migration

**DOI:** 10.1371/journal.pone.0089292

**Published:** 2014-02-21

**Authors:** Masanari Umemura, Erdene Baljinnyam, Stefan Feske, Mariana S. De Lorenzo, Lai-Hua Xie, Xianfeng Feng, Kayoko Oda, Ayako Makino, Takayuki Fujita, Utako Yokoyama, Mizuka Iwatsubo, Suzie Chen, James S. Goydos, Yoshihiro Ishikawa, Kousaku Iwatsubo

**Affiliations:** 1 Cardiovascular Research Institute, Yokohama City University School of Medicine, Yokohama, Japan; 2 Department of Cell Biology and Molecular Medicine, New Jersey Medical School, Rutgers, The State University of New Jersey, Newark, New Jersey, United States of America; 3 Department of Pathology, New York University School of Medicine, New York, New York, United States of America; 4 Department of Chemical Biology, Susan Lehman Cullen Laboratory of Cancer Research in the Ernest Mario School of Pharmacy, Rutgers, The State University of New Jersey, Piscataway, New Jersey, United States of America; 5 Division of Surgical Oncology, Department of Surgery, Robert Wood Johnson Medical School, Rutgers, The State University of New Jersey, Piscataway, New Jersey, United States of America; University of Debrecen, Hungary

## Abstract

Store-operated Ca^2+^ entry (SOCE) is a major mechanism of Ca^2^^+^ import from extracellular to intracellular space, involving detection of Ca^2+^ store depletion in endoplasmic reticulum (ER) by stromal interaction molecule (STIM) proteins, which then translocate to plasma membrane and activate Orai Ca^2+^ channels there. We found that STIM1 and Orai1 isoforms were abundantly expressed in human melanoma tissues and multiple melanoma/melanocyte cell lines. We confirmed that these cell lines exhibited SOCE, which was inhibited by knockdown of STIM1 or Orai1, or by a pharmacological SOCE inhibitor. Inhibition of SOCE suppressed melanoma cell proliferation and migration/metastasis. Induction of SOCE was associated with activation of extracellular-signal-regulated kinase (ERK), and was inhibited by inhibitors of calmodulin kinase II (CaMKII) or Raf-1, suggesting that SOCE-mediated cellular functions are controlled via the CaMKII/Raf-1/ERK signaling pathway. Our findings indicate that SOCE contributes to melanoma progression, and therefore may be a new potential target for treatment of melanoma, irrespective of whether or not Braf mutation is present.

## Introduction

Melanoma has the poorest prognosis among skin cancers, although drugs targeting aberrant ERK signaling, i.e., mutated serine/threonine-protein kinase Braf, have improved both overall and progression-free survival times [1]. However, this therapy is not effective in patients without Braf mutation, and some patients with Braf mutation rapidly acquire resistance to Braf inhibitors [2]. Accordingly, a different approach to target ERK signaling regardless of Braf mutation is needed.

Intracellular Ca^2^^+^ signaling regulates diverse cellular functions including proliferation and cell migration [3]. Store-operated Ca^2+^ entry (SOCE) is a major mechanism of Ca^2+^ import from extracellular to intracellular space, especially in non-excitable cells [4]. In general, activation of inositol 1,4,5-trisphosphate (IP_3_) receptors on the endoplasmic reticulum (ER) evokes a rapid and transient release of Ca^2+^ from the ER store. The resulting decrease of Ca^2^^+^ concentration in the ER is sensed by the EF-hand motif of stromal interaction molecules (STIM), which then translocate to the plasma membrane, where they interact with Orai Ca^2+^ channel subunits [5], leading to Ca^2+^ influx from extracellular space to restore the Ca^2+^ concentration in ER [6].

The physiological functions of STIM and Orai have been studied mainly in connection with the immune system [7]–[10]. Orai channels control Ca^2+^ release-activated Ca^2+^ (CRAC) currents in lymphocytes [4], and also contribute to SOCE currents in other types of cells, such as endothelial cells [11]. STIM1 and Orai1, but not STIM2, Orai2 or Orai3, have roles in cell migration of smooth muscle cells [12], [13]. Examination of a library of randomized ribozymes indicated that STIM1 is a metastasis-related gene [14]. SOCE is involved in proliferation, cell migration, and angiogenesis in cervical cancer [15], and cell migration in breast cancer [16]. However, the role of SOCE in melanoma has been little investigated, except for a recent paper demonstrating Akt signaling activation in mouse melanoma cells, especially in lipid rafts [17]. In the present study, we show that SOCE promotes melanoma progression by enhancing cell proliferation, migration, and metastasis through activation of ERK signaling via the CaMKII/Raf-1/ERK pathway.

## Materials and Methods

### Reagents and Cell Lines

Reagents were purchased from Sigma unless otherwise specified. Antibodies to β-actin, GAPDH, and ERK were purchased from Santa Cruz. α-Spectrin antibody was purchased from Millipore. Phospho-ERK antibody was purchased from Cell Signaling. Antibodies against STIM1 were purchased from BD Transduction Laboratories and Abnova [18]. Antibodies against Orai1 were previously generated by us [19], or purchased from Sigma [12]. Second antibodies for mouse and rabbit were purchased from Abcam and Cell Signaling, respectively. GW5074 was purchased from Focus Biomolecules. W5 hydrochloride was purchased from Tokyo Chemical Industry. GDC-0879 was purchased from Selleckhem [20]. SK-Mel-2 and SK-Mel-24 (human metastatic melanoma) cell lines were obtained from the American Type Culture Collection. UACC257 (human metastatic melanoma) was obtained from the Charles River Laboratory. Melan-A mouse melanocyte cell line was purchased from Welcome Trust Functional Genomics Cell Bank, St. George’s, University of London. C8161 cell line was kindly provided by Dr. Mary J.C. Hendrix. WM3248 and WM115 (primary melanoma, vertical growth phase (VGP)) and WM1552C (primary melanoma, radial growth phase (RGP)) cell lines were kindly provided by Dr. Meenhard Herlyn. HEMA-LP (human melanocyte) cell line was obtained from Invitrogen. SK-Mel-2 and SK-Mel-24 cells were maintained in MEM containing 10% fetal bovine serum (FBS) and 1% penicillin-streptomycin. UACC257 cells were maintained in RPMI-1640 (Sigma) containing 10% FBS and 1% penicillin-streptomycin. HEMA-LP was maintained in an EndoGRO-VEGF Complete media kit (Millipore). All other melanoma cells were maintained in RPMI (Gibco) containing 10% FBS and 1% penicillin-streptomycin.

### Western Blot Analysis

Western blot analyses were performed as we previously described [21]. Briefly, cells were lysed and sonicated in RIPA buffer (Thermo Scientific). Equal amounts of protein were subjected to sodium dodecyl sulfate polyacrylamide gel electrophoresis (SDS-PAGE). After protein separation by electrophoresis, samples were transferred to Millipore Immobilon-P membrane followed by immunoblotting with antibodies against molecules of interest. Immunoblottings for STIM1 and Orai1 were performed with the antibodies from BD Transduction Laboratories and from Sigma, respectively. Signal intensities of bands were quantified with Image J software (NIH).

### Immunohistochemistry

Immunohistochemical stainings were performed as we previously described [22]. Melanoma tissue microarray plates (US Biomax Cat. #T085 and #ME1004a) were subjected to immunohistochemistry with antibodies against melanoma antigen recognized by T-cells 1 (MART1) (Millipore), STIM1 and Orai1. Two different antibodies against STIM1 (purchased from BD Transduction Laboratories and Abnova) and Orai1 (generated by us [19] and purchased from Sigma) were used to confirm the specificity of the staining. Negative control samples were exposed to the secondary antibody alone. Quantification of STIM1 expression was performed with BZ-II analyzer software (Keyence) as we previously described [23].

### Fluorescence Imaging of Intracellular Ca^2+^

Measurement of intracellular Ca^2+^ level was performed as we previously described [24]. Cells were incubated with 2-[4-(2-hydroxyethyl)-1-piperazinyl]ethanesulfonic acid (HEPES) buffer containing 4 µmol/l of Fluo-4AM, followed by washing and incubation with HEPES-buffered saline containing 2.0 mmol/L of CaCl_2_. An iXon+885 charge-coupled-device camera (Andor Technology) was used to monitor fluorescence changes. Full images were collected every 4 s. Fluo-4 fluorescence was excited at 488 nm, and data were expressed as normalized changes in background-corrected fluorescence emission (F/F_0_). Data were analyzed using Imaging Workbench (INDEC BioSystems). Representative Ca^2+^ signals averaged from 6 to 10 individual cells are shown in the figures.

### Transduction of Short Hairpin RNA (shRNA)

SK-Mel-2, SK-Mel-24 and C8161 cells were transduced with STIM1 shRNA, Orai1 shRNA, and scramble control shRNA using lentivirus (Santa Cruz Biotechnology) according to the protocols provided by the manufacturer. Briefly, cells were incubated with 10 µg/mL of Polybrene (Santa Cruz Biotechnology) and lentiviral particles harboring each shRNA, then selected with puromycin dihydrochloride (Santa Cruz Biotechnology) for 1 week. Puromycin-containing medium was replaced with fresh medium every 3 to 4 days.

### 3-(4,5-Dimethylthiazol-2-yl)-2,5-diphenyltetrazolium Bromide (MTT) Assay

Cells were seeded in a 96-well plate at 5,000 or 10,000 cells per well and cultured for 24 h. Viable cells were determined daily using the MTT Cell Proliferation Assay kit (ATCC) according to the manufacturer’s instructions.

### Apoptosis Assays

Apoptosis assays were performed as previously described [25]. Cells were seeded on 6 cm dishes, and incubated for 24 or 48 hours. Cells were washed twice with cold PBS, and transferred into culture tubes. Annexin V, allophycocyanin conjugate (APC) and 7-amino-actinomycin D (7-AAD) (BD Biosciences, California, U.S.A.) were then added to the tubes. Cells were incubated for 15 min at RT (25°C) in darkness, followed by FACS analysis (Canto™ II, Japan Becton, Dickinson and Company, Tokyo, Japan) within 1 hour.

### Migration Assays

Migration assays were performed using 24-well Boyden chambers (8 µm pores, BD Biosciences) as we previously described [25]. The cells were plated at a density of 1×10^5^ cells/100 µl of medium in the inserts, and incubated for 3 h at 37°C, followed by staining using a Diff-Quick kit (SIMENS). Pictures were taken with a microscope to count the number of migrated cells. The scratch wound method was also employed in some experiments as follows. Cells were seeded and allowed to form a monolayer for 24 h. The dish was scraped with the tip of a 100 µl pipette, and the resulting wound was washed with PBS two times [12]. Culture medium containing 10% FBS was added to the cells, and the cells were incubated at 37°C in 5% CO_2_. Bright-field images were captured (Olympus IX51 microscope) and analyzed (Adobe Photoshop). The total number of pixels in empty spaces inside the wound were counted and normalized to the control.

### Time-lapse Videomicroscopy

Analysis of cell motility using time-lapse videomicroscopy was performed as we previously described [25]. SK-Mel-2 cells were subjected to time-lapse video recording. Frames from the recording were digitized at 15-min intervals. Moving distance of each cell was analyzed by Image J software (NIH).

### Calpain Activity Assay

Calpain activity was performed as previously described [26] with the calpain activity kit (Abcam) according to the manufacturer’s instructions.

### Immunocytochemistry

Immunocytochemistry was performed as previously described [24]. Filamentous actin (F-actin) staining was performed by incubation with rhodamine phalloidin (Invitrogen). Photographs were taken with a digital camera on a Nikon Eclipse TE200, and cells with one lamellipodium or more were counted manually.

### Lung Colonization Assay

Lung colonization assay was performed as we previously described [22]. Cells were harvested and injected (2×10^6^ cells/0.2 ml) into the tail veins of BALB/c nude mice (Charles River, male, 8 weeks old). Three weeks after the injection, metastatic colonies on the surface of the lungs were fixed with picric acid and counted under a dissection microscope.

### Data Analysis and Statistics

Statistical comparisons among groups were performed using Student’s *t*-test or one-factor analysis of variance (ANOVA) with the Bonferroni post hoc test. The criterion of statistical significance was set at *p*<0.05. *, *p*<0.05, **, *p*<0.01, N.S., not significant.

### Ethics Statement

All animal studies were approved by the Institutional Animal Care and Use Committees of New Jersey Medical School-Rutgers, The State University of New Jersey (Protocol Number: 11104D0914).

## Results

### STIM1 and Orai1 are Expressed in Human Melanoma

**Figure 1:**
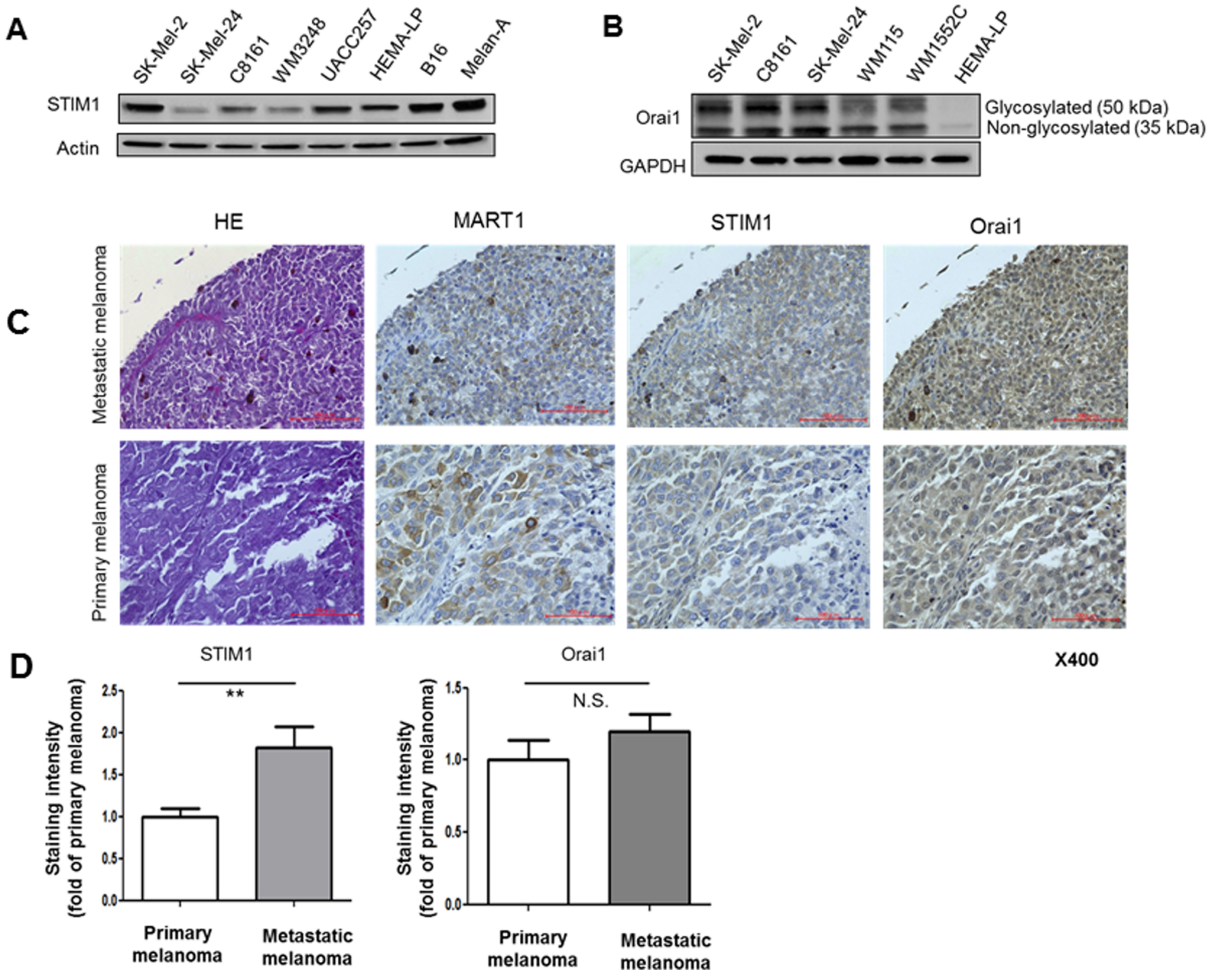
Expression of STIM1 and Orai1 in melanoma. (**A and B**) Western blot analyses of STIM1 (A) and Orai1 (B) expression in the indicated cell lines. (**C**) Immunohistochemical stainings of HE, MART1, STIM1 (the antibody from Abnova was used) and Orai1 (the antibody from Sigma was used) in a melanoma tissue microarray (original magnification, x400). Calibration bars represent 100 µm. (**D**) Analyses of the staining intensity of expression of STIM1 and Orai1. *, *p*<0.05; **, *p*<0.01, n = 8.

We first examined expression of STIM and Orai in human melanoma. Western blot analyses showed that STIM1 and Orai1 are expressed in metastatic human melanoma cell lines, while the melanocyte cell line, HEMA-LP, displayed only a low level of Orai1 expression (Fig. 1A and B). In contrast, STIM2, Orai2 and Orai3 expression was undetectable or barely detectable in the melanoma cell lines (data not shown). We also examined expression of STIM1 and Orai1 in human melanoma tissues, using a microarray. Both molecules were immunohistochemically detected (Fig. 1C). Interestingly, STIM1, but not Orai1, showed higher expression in metastatic melanoma than in primary melanoma (Fig. 1D). This finding was confirmed using a different set of antibodies. These data suggested that STIM1 expression, but not Orai1 expression, positively correlates with melanoma progression.

### SOCE Occurs in Melanoma Cell Lines

**Figure 2:**
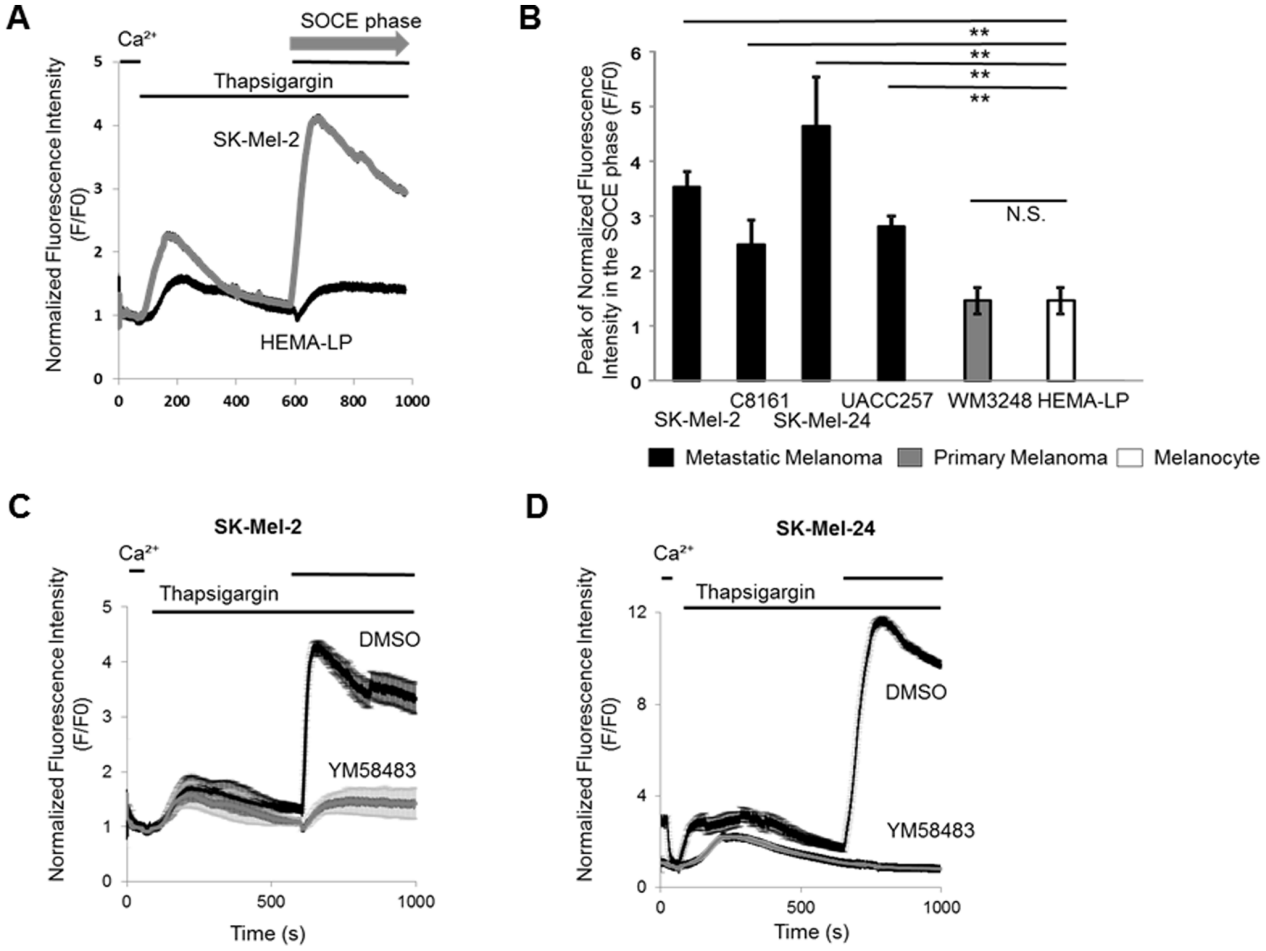
SOCE occurs in melanoma. (**A**) Cytosolic Ca^2+^ levels in SK-Mel-2 cells. Extracellular Ca^2+^ (2 mM) was removed, followed by the addition of thapsigargin (TG) (2 µM) for Ca^2+^ depletion in the ER. Ca^2+^ (2 mM) was then added to the extracellular fluid, and SOCE-induced Ca^2+^ elevation was observed (SOCE phase). (**B**) Ca^2+^ peak amplitude in the SOCE phase was compared among melanoma and melanocyte cell lines. **, *p*<0.01 (HEMA-LP), n = 6–8. (**C and D**) Cytosolic Ca^2+^ levels in SK-Mel-2 and SK-Mel-24 cells are shown as means ± SD (n = 6–10). SOCE was examined in the presence or absence of DMSO (1 µM) or YM58483 (1 µM) in SK-Mel-2 (**C**) and SK-Mel-24 cells (**D**).

Since STIM1 and Orai1 were detected in human melanoma tissues, we examined whether SOCE occurs in melanoma cell lines. SOCE is defined as enhanced Ca^2+^ import from extracellular space after depletion of Ca^2+^ stores in the ER. Experimentally, SOCE is induced by Ca^2+^ addition after Ca^2+^ depletion from the ER with thapsigargin (Fig. 2A), an inhibitor of sarcoplasmic reticulum Ca^2+^-ATPase (SERCA). We observed SOCE in some melanoma and melanocyte cell lines examined (Fig. 2B). Metastatic (SK-Mel-2, C8161, SK-Mel-24 and UACC2577), but not primary (WM3248, WM115 (data not shown) and WM1552C (data not shown)), melanoma cell lines showed higher SOCE peak amplitudes than the melanocyte cell line (HEMA-LP). These data suggested that SOCE is enhanced in metastatic melanoma, in accordance with the idea that activation of SOCE is related to melanoma progression. A pyrazole compound, YM58483, which is known to inhibit SOCE in non-melanoma cells [27], [28], also suppressed SOCE in metastatic melanoma cell lines (Fig. 2C and D).

### STIM1- or Orai1-knockdown Inhibits SOCE in Melanoma Cell Lines

**Figure 3:**
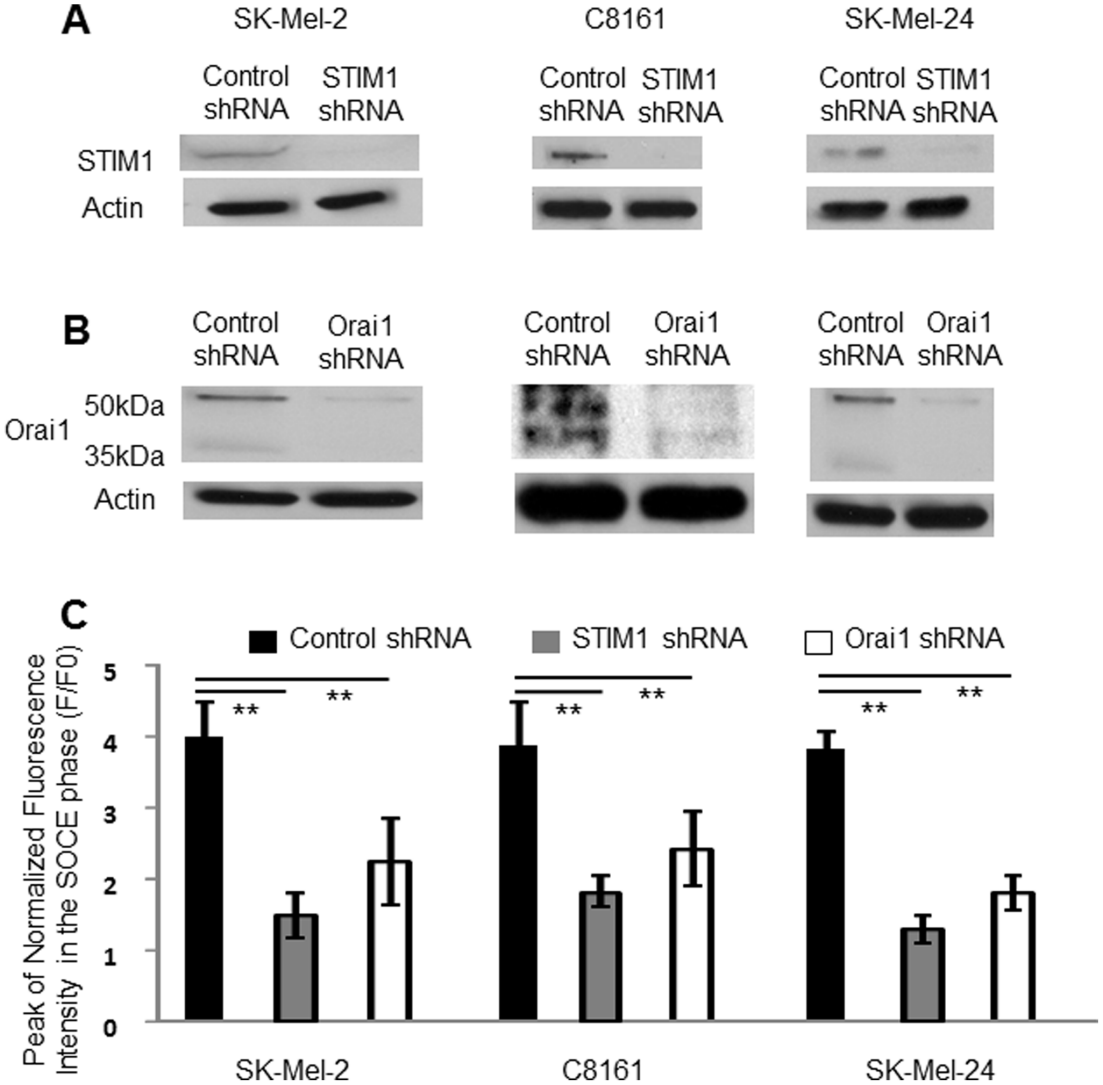
SOCE was inhibited by STIM1- or by Orai1-knockdown in melanoma cell lines. (**A and B**) Western blot analyses of protein expressions of STIM1 (A) and Orai1 (B) in metastatic melanoma cell lines. shRNA transduction reduced expression of the target proteins. (**C**) Ca^2+^ peak amplitude of SOCE was reduced by STIM1- or Orai1-knockdown. **, *p*<0.01, n = 6–10.

We examined whether SOCE in melanoma cell lines is regulated by STIM1 and Orai1. Ca^2+^ peak amplitude in the SOCE phase was lower in both STIM1- (Fig. 3A) and Orai1- (Fig. 3B) knockdown cells than in control metastatic melanoma cell lines (Fig. 3C). These data suggested that both STIM1 and Orai1 are involved in SOCE activation in melanoma.

### Inhibition of SOCE Suppresses Melanoma Cell Proliferation

**Figure 4:**
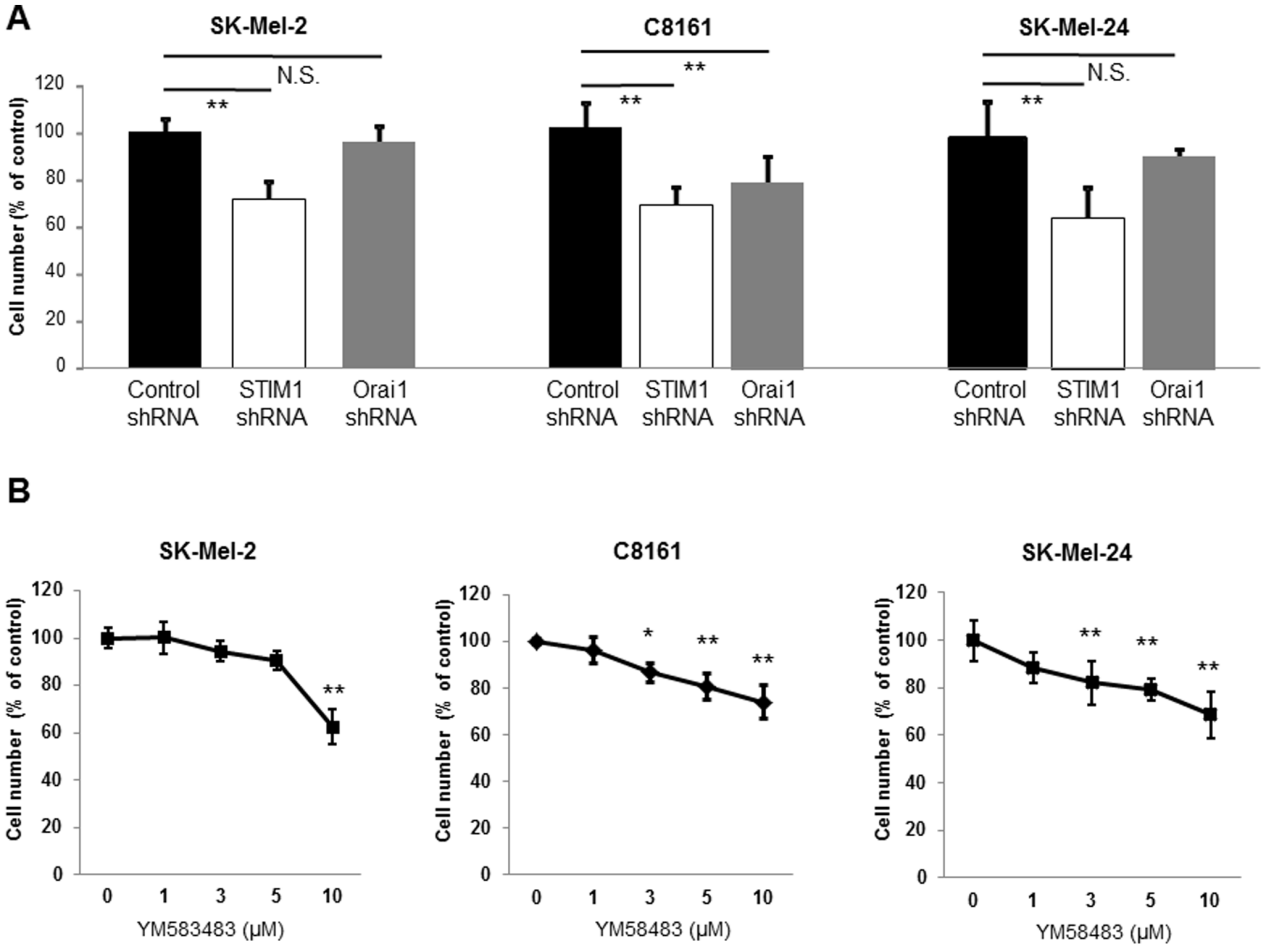
SOCE regulates proliferation of melanoma cells. (**A**) MTT assay of metastatic melanoma cell lines with or without STIM1- or Orai1-knockdown. **, *p*<0.01, n = 8. Representative data of 3 independent experiments are shown. (**B**) MTT assay of the indicated cell lines in the presence or absence of YM58483. *, *p*<0.05; **, *p*<0.01, n = 8. Representative data of 3 independent experiments are shown.

We next examined the role of SOCE in cellular functions of melanoma. We found that proliferation was reduced in STIM1-knockdown metastatic melanoma cell lines (Fig. 4A). This was not due to induction of apoptosis, because there was no significant difference of apoptosis between STIM1-knockdown and control C8161 cells after both 24 hours and 48 hours (Fig. S1). In contrast, Orai1-knockdown inhibited proliferation in C8161 cells but not in SK-Mel-2 or SK-Mel-24 cells, suggesting that STIM1, rather than Orai1, is the key determinant of melanoma proliferation. The SOCE inhibitor YM58483 (Fig. 4B) inhibited proliferation of SK-Mel-2, C8161 and SK-Mel-24 cells, further supporting the view that SOCE plays an important role in melanoma proliferation.

### Inhibition of SOCE Suppresses Melanoma Cell Migration/Metastasis

**Figure 5:**
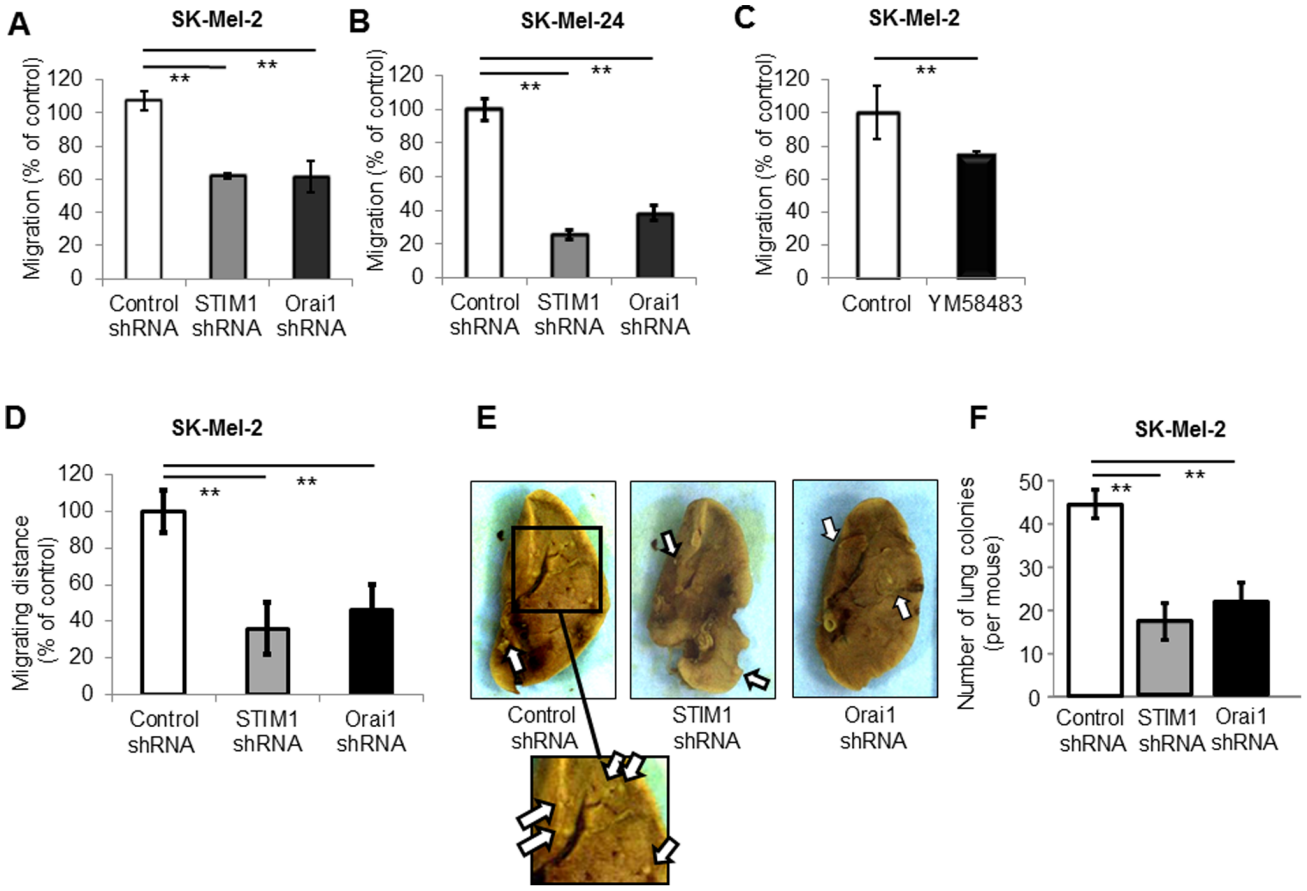
SOCE regulates cell migration and metastasis of melanoma. (**A and B**) Boyden chamber assay showed that either STIM1- or Orai1-knockdown inhibited cell migration. **, *p*<0.01, n = 4. (**C**) The scratch test showed that YM583483 (1 µM) inhibited cell migration. **, *p*<0.01, n = 4. (**D**) Time-lapse video recording (Videos S1, S2, and S3) showed that STIM1- or Orai1-knockdown SK-Mel-2 cells exhibited a shorter migration distance than control SK-Mel-2 cells. **, *p*<0.01, n = 10. (**E and F**) SK-Mel-2 cells with knockdown of either STIM1 or Orai1 were injected to the tail vein of Balb/c nu/nu mice. Three weeks later, the lungs were removed and fixed with picric acid. (**E**) Representative images of lung surface are shown. Arrows indicate metastatic melanoma colonies (white lesions). (**F**) The number of metastatic colonies in the lung surface was counted under a dissection microscope. **, *p*<0.01, n = 8.

Since Ca^2^^+^ signaling regulates migration of cancer cells [29], we also examined the role of SOCE in melanoma cell migration. We found that both STIM1- and Orai1-knockdown inhibited cell migration in metastatic melanoma cell lines (Fig. 5A and B). YM58483 also inhibited melanoma cell migration (Fig. 5C). Time-lapse video recordings showed reduced cell migration distance of both STIM1- and Orai1-knockdown cells (Fig. 5D and Videos S1, S2, and S3). We next examined whether the reduced cell migration is correlated with inhibition of metastasis. Both STIM1- and Orai1-knockdown resulted in decreased numbers of metastatic colonies in the lungs of mice (Fig. 5E and F). These data suggested that the inhibition of SOCE suppresses melanoma cell migration and thereby reduces metastasis.

### SOCE Regulates ERK Signaling in Melanoma Cell Lines

**Figure 6:**
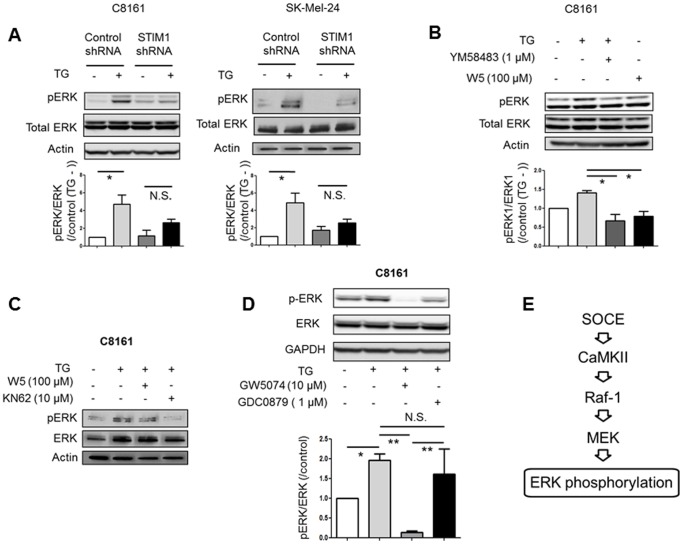
SOCE increases phosphorylation of ERK in melanoma. (**A**) Densitometric analyses (bar graph) of western blots show that thapsigargin increased phosphorylation of ERK1/2 in metastatic melanoma cell lines. STIM1-knockdown inhibited thapsigargin-induced phosphorylation of ERK1/2. *, *p*<0.05; N.S., not significant, n = 3. (**B**) Densitometric analyses (bar graph) of western blots show that thapsigargin-induced phosphorylation of ERK1/2 was inhibited by SOCE inhibitor YM58483 and CaM inhibitor W5 in C8161 cells. *, *p*<0.05, n = 3. (**C**) Thapsigargin-induced phosphorylation of ERK1/2 was inhibited by CaM inhibitor W5 and CaMKII inhibitor KN62 in C8161 cells. (**D**) Densitometric analyses (bar graph) of western blots show that thapsigargin-induced phosphorylation of ERK1/2 was inhibited by Raf-1 inhibitor GW5074, but not by Braf inhibitor GDC0879. *, *p*<0.05; **, *p*<0.01; N.S. not significant, n = 3. (**E**) Proposed signaling schema of SOCE-induced ERK activation.

The ERK signaling pathway is known to play a major role in melanoma cells migration and proliferation [30], [31]. Thus, we next examined whether SOCE affects ERK signaling. Phosphorylation of ERK1/2, which reflects activity of ERK signaling, was increased by thapsigargin in metastatic melanoma cell lines (Fig. 6A). The thapsigargin-induced ERK1/2 phosphorylation was attenuated by STIM1-knockdown (Fig. 6A) and by YM58483 (Fig. 6B), suggesting that thapsigargin-induced ERK activation occurs via a SOCE-related mechanism. Since it has been demonstrated that intracellular Ca^2+^ activates ERK signaling via calmodulin (CaM)/calmodulin-dependent protein kinase II (CaMKII) [32], we examined the involvement of these molecules in the SOCE-induced ERK activation. Thapsigargin-induced ERK1/2 phosphorylation was inhibited by W5, a CaM inhibitor (Fig. 6B and C), and by KN62, a CaMKII inhibitor (Fig. 6C), suggesting that SOCE-induced ERK activation is mediated by CaM/CaMKII. Thapsigargin-induced ERK1/2 phosphorylation was inhibited by GW5074, a specific Raf-1 inhibitor, but not by GDC0879, a specific Braf inhibitor, in C8161 cells (Fig. 6D). This is in accordance with previous findings that CaMKII can bind to and activate Raf-1 [33], [34]. Similar results were also observed in SK-Mel-24 cells (data not shown), suggesting that Raf-1, rather than Braf, predominantly mediates SOCE-mediated ERK activation (Fig.6E). These data suggested that the conventional ERK signaling pathway is activated by SOCE via CaM/CaMKII.

### SOCE Regulates Melanoma Cell Migration via ERK Signaling

**Figure 7:**
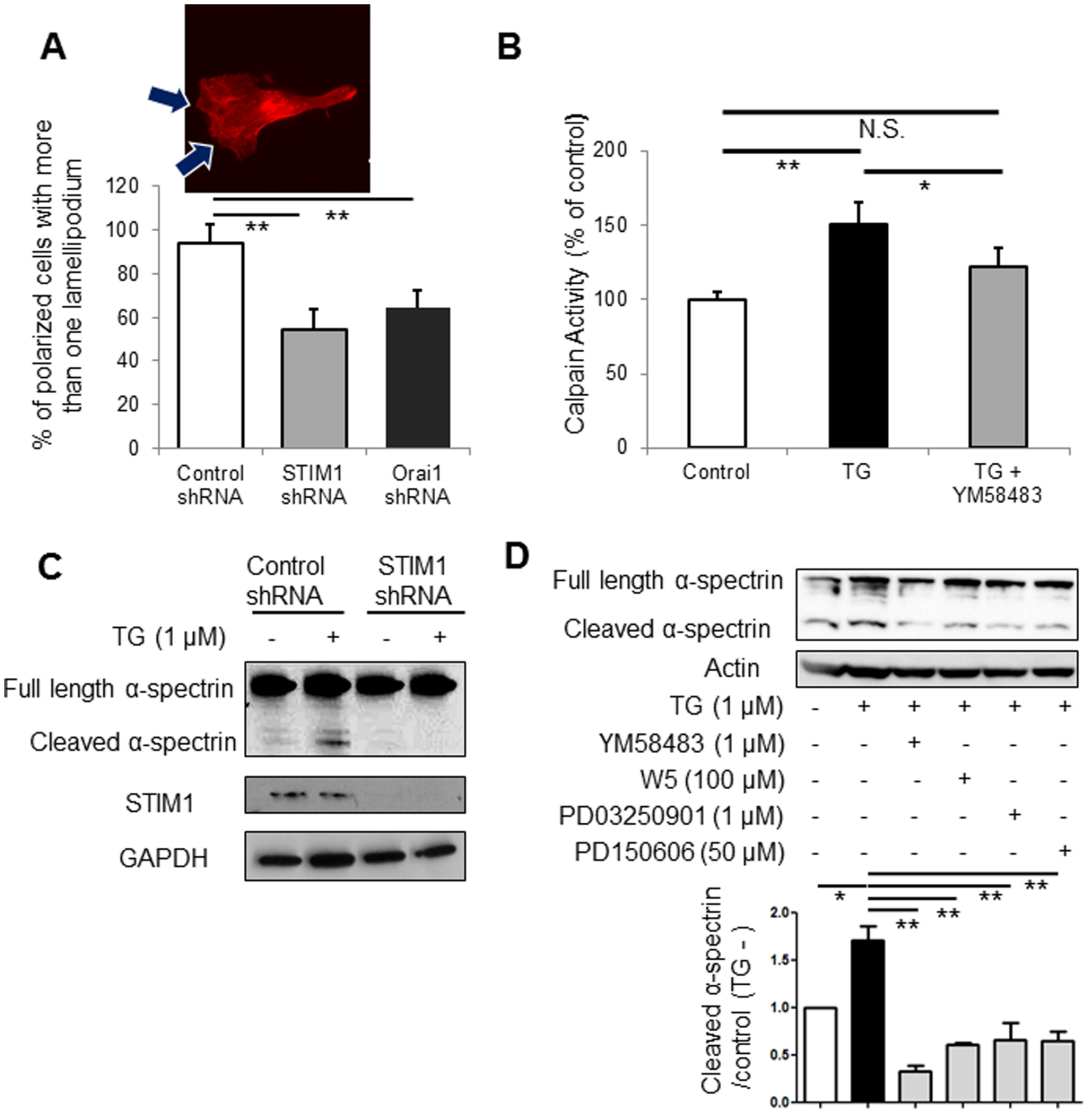
Proposed mechanisms of SOCE-mediated cellular functions in melanoma. (**A**) The upper panel shows actin staining of SK-Mel-2 cells. Arrows indicate lamellipodia. The lower panel shows the number of SK-Mel-2 cells with more than one lamellipodium. **, *p*<0.01, n = 331 for control shRNA, 850 for STIM1 shRNA, and 986 for Orai1 shRNA. (**B**) Calpain activity assay shows that thapsigargin increased calpain activity. Thapsigargin-induced elevation of calpain activity was inhibited by YM58483. *, *p*<0.05, **, *p*<0.01, N.S., not significant, n = 4. (**C**) Western blot analyses showed that thapsigargin (TG) increased cleavage of α-spectrin in SK-Mel-2 cells. Thapsigargin-induced cleavage of α-spectrin was inhibited by STIM1-knockdown. Representative data of 3 independent experiments are shown. (**D**) Densitometric analyses (bar graph) of western blots show that thapsigargin-induced cleavage of α-spectrin was inhibited by SOCE inhibitor YM58483, CaM inhibitor W5, MEK inhibitor PD03250901, and calpain inhibitor PD150606. *, *p*<0.05; **, *p*<0.01, n = 3.

There have been extensive studies on the mechanism of melanoma cell proliferation via ERK signaling [35], [36]. However, the molecular mechanism through which ERK signaling regulates melanoma cell migration remains relatively unclear. Intracellular Ca^2+^ accelerates cell migration via calpain, a proteolytic enzyme, which promotes actin assembly/disassembly [37], [38]. Therefore, it was suggested that SOCE regulates melanoma cell migration via calpain-dependent actin dynamics. This hypothesis was supported by the observation that the number of lamellipodia, which reflects the activity of actin assembly/disassembly [39], [40], was decreased by STIM1- or Orai1-knockdown (Fig. 7A). In addition, we found that calpain activity was increased by thapsigargin, but was inhibited by YM58483 (Fig. 7B). Further, cleavage of α-spectrin, a target enzyme of calpain, was increased by thapsigargin, but was inhibited by STIM1-knockdown (Fig. 7C). These data suggested that SOCE regulates cell migration in a calpain-dependent manner. The question of how SOCE activates calpain then arises. Calpain was originally found as a Ca^2+^-dependent enzyme [41], but recently it was demonstrated that ERK signaling can increase calpain activity [42]–[44]. We thus examined whether ERK signaling rather than intracellular Ca^2+^ itself modulates calpain activity. As shown in Fig. 7D, thapsigargin-induced α-spectrin cleavage was inhibited by YM58483, and by PD150606, a calpain inhibitor, as expected. In addition, inhibition of CaM by W5 and inhibition of MEK by PD03250901 suppressed thapsigargin-induced α-spectrin cleavage. These data suggested that calpain mediates SOCE-induced cell migration via ERK signaling, not via simple SOCE-induced elevation of intracellular Ca^2+^.

## Discussion

Our present findings show that SOCE occurs in melanoma cells and plays a pivotal role in cell proliferation and migration, most probably via ERK signaling. STIM1 and Orai1 were expressed not only in cultured melanoma cells, but also in human melanoma tissues. SOCE activity in melanoma cells was dependent on STIM1 and Orai1, and was reduced by SOCE inhibitor YM58483. Inhibition of SOCE suppressed both melanoma proliferation and cell migration. In addition, SOCE activated ERK signaling in melanoma cells, which may lead to further changes in cellular functions. Accordingly, our results suggest that melanoma progression is promoted by SOCE via ERK signaling. Recent melanoma therapies have targeted V600E-mutated Braf [1], but some patient are non-responders, and acquisition of resistance is also a problem [2]. Our data showed that SOCE occurs not only in melanoma cells bearing Braf-mutation (SK-Mel-24, UACC257, WM3248, WM115, and WM1552C), but also in non-Braf-mutation-carrying melanoma cells lines (SK-Mel-2 and C8161). In addition, we found that STIM1-knockdown inhibited ERK phosphorylation in both Braf-mutated (SK-Mel-24) and non-Braf-mutated (SK-Mel-2 and C8161) cells. These data suggested that inhibition of SOCE suppresses ERK signaling activity irrespective of the existence of Braf mutation. Therefore, targeting SOCE could potentially benefit a greater number of melanoma patients, compared to currently used Braf inhibitors.

The role of SOCE in proliferation and cell migration is well established. Abdullaev *et al*. showed that STIM1 and Orai1 regulate CRAC currents and SOCE, leading to changes in proliferation of endothelial cells [11]. In vascular smooth muscle cells, knockdown of either STIM1 or Orai1, but not of STIM2, Orai2, and Orai3, inhibited proliferation and cell migration [13]. In cancer cells, Yang *et al.* reported that STIM1-knockdown inhibited serum-induced breast tumor cell migration [16]. The same group also showed that STIM1-knockdown in hepatocarcinoma cells affects disassembly and turnover of focal adhesion [45]. Our data demonstrate that STIM1 and Orai1 have roles in proliferation and migration of various melanoma cell lines, indicating that SOCE contributes to melanoma progression. Orai1 knockdown had only a minor effect on proliferation compared to STIM1 knockdown, suggesting that TRPC channels rather than Orai1 potentially interact with STIM [46] in the regulation of proliferation. There was a discrepancy between the effective concentrations of YM58483 for inhibition of proliferation and inhibition of SOCE. This can be attributed, at least in part, to compensatory upregulation of SOCE [47], which would occur during the relatively prolonged exposure to the SOCE-inhibitory drug in MTT assay compared to the short exposure during SOCE measurement. Another concern is the fact that Orai1 deletion did not inhibit proliferation of SK-Mel-2 and SK-Mel-24 cells, whereas YM58483 treatment did. This might be explained by incomplete knockdown of Orai1.

It was recently demonstrated that, in mouse melanoma cells, SOCE occurs in lipid rafts, and ablation of the rafts suppressed tumor growth, most probably via the Akt pathway [48]. These data, taken together with ours, indicate that SOCE regulates multiple pathways in melanoma, supporting our proposal that SOCE plays a pivotal role in melanoma progression.

The upstream pathway leading to SOCE in melanoma cells remains unknown. Ca^2+^ depletion in the ER is mainly controlled by the phospholipase C (PLC)/IP_3_/IP_3_ receptor pathway, which is generally activated by either tyrosine kinase-type receptors or Gq-related G protein coupled receptors. Previous reports indicated that SOCE is controlled by tyrosine kinase-type receptors rather than Gq-coupled receptors. For example, in colorectal cancer cells, epidermal growth factor (EGF) increased expression of cyclooxygenase-2 via STIM1 and Orai1 [49]. In pulmonary arterial smooth muscle cells, platelet-derived growth factor (PDGF) activated SOCE via Akt signaling [50]. In addition, both EGF [51] and PDGF [52] can increase the activity of ERK signaling in melanoma. It will be interesting to investigate the functional relationship among tyrosine kinase-type receptors, SOCE and the ERK signaling in melanoma cells.

In conclusion, our results indicate that inhibition of SOCE suppressed proliferation and cell migration/metastasis of melanoma cells, most probably via ERK signaling. We propose that SOCE is a potential target for treatment of melanoma, irrespective of whether or not Braf mutation is present.

## Supporting Information

Figure S1 **STIM1-knockdown did not promote apoptosis.** APC Annexin V and 7-AAD staining assays demonstrated that apoptosis of STIM1-knockdown and control shRNA-transduced C8161 cells was not different. These data demonstrate that apoptosis did not contribute to the STIM1-knockdown-induced inhibition of cell proliferation. N.S. not significant, n = 4.
(TIF)

Video S1 **Melanoma cell migration was inhibited by STIM1- or by Orai1-knockdown.** Cellular movement was recorded by time-lapse video microscopy recording. Video S1, control shRNA, video S2, STIM1 shRNA, video S3, Orai1 shRNA.
(WMV)

Video S2 **Melanoma cell migration was inhibited by STIM1- or by Orai1-knockdown.** Cellular movement was recorded by time-lapse video microscopy recording. Video S1, control shRNA, video S2, STIM1 shRNA, video S3, Orai1 shRNA.
(WMV)

Video S3 **Melanoma cell migration was inhibited by STIM1- or by Orai1-knockdown.** Cellular movement was recorded by time-lapse video microscopy recording. Video S1, control shRNA, video S2, STIM1 shRNA, video S3, Orai1 shRNA.
(WMV)
